# An outbreak of Shiga toxin-producing *Escherichia coli* (STEC) O157:H7 associated with contaminated lettuce and the cascading risks from climate change, the United Kingdom, August to September 2022

**DOI:** 10.2807/1560-7917.ES.2024.29.36.2400161

**Published:** 2024-09-05

**Authors:** Neil Cunningham, Claire Jenkins, Sarah Williams, Joanna Garner, Bernd Eggen, Amy Douglas, Tina Potter, Anthony Wilson, Giovanni Leonardi, Lesley Larkin, Susan Hopkins

**Affiliations:** 1 United Kingdom Health Security Agency (UKHSA), London, United Kingdom; 2 United Kingdom Field Epidemiology Training Programme, United Kingdom Health Security Agency (UKHSA), London, United Kingdom; 3 Food Standards Agency (FSA), London, United Kingdom

**Keywords:** One Health, STEC, E. coli, Climate change, Outbreak investigation, Surveillance

## Abstract

Shiga-toxin producing *Escherichia coli* (STEC) O157 is a food-borne pathogen which causes gastrointestinal illness in humans. Ruminants are considered the main reservoir of infection, and STEC exceedance has been associated with heavy rainfall. In September 2022, a large outbreak of STEC O157:H7 was identified in the United Kingdom (UK). A national-level investigation was undertaken to identify the source of the outbreak and inform risk mitigation strategies. Whole genome sequencing (WGS) was used to identify outbreak cases. Overall, 259 cases with illness onset dates between 5 August and 12 October 2022, were confirmed across the UK. Epidemiological investigations supported a UK grown, nationally distributed, short shelf-life food item as the source of the outbreak. Analytical epidemiology and food chain analysis suggested lettuce as the likely vehicle of infection. Food supply chain tracing identified Grower X as the likely implicated producer. Independent of the food chain investigations, a novel geospatial analysis triangulating meteorological, flood risk, animal density and land use data was developed, also identifying Grower X as the likely source. Novel geospatial analysis and One Health approaches are potential tools for upstream data analysis to predict and prevent contamination events before they occur and to support evidence generation in outbreak investigations.

**Key public health message**

**What did you want to address in this study and why?**

Shiga-toxin producing *E. coli* (STEC) O157 are bacteria that can be found in faeces of animals such as cattle and sheep. When STEC infect humans, they can cause food poisoning and severe illness, with some people requiring hospital admission. In September 2022, a large outbreak of STEC was identified in the United Kingdom with 259 cases. We wished to identify the cause of the outbreak and understand if climatic factors played a role.

**What have we learnt from this study?**

We identified contaminated lettuce as the likely vehicle of infection in the outbreak by using whole genome sequencing, interviewing cases and tracing the food back through the supply chain. Using new techniques, we were able to use weather data (rainfall and temperature), information about how land is used and information about the location of sheep to better understand the events that led to the outbreak and the location of the lettuce grower.

**What are the implications of your findings for public health?**

Climate change will have increasing impacts on our health and food security. We expect to see more heavy rainfall events. The lettuce incriminated in this outbreak may have been contaminated by heavy rainfall and flooding, transporting STEC from animal faeces to crops in fields. Our new techniques could help to predict and prevent future outbreaks and inform risk assessments and risk management for farmers growing fresh produce for people to eat.

## Background

Shiga toxin-producing *Escherichia coli* (STEC) serotype O157 is a food-borne gastrointestinal pathogen of public health concern. Infections with STEC can present as sporadic cases or as outbreaks, and they can be symptomatic or asymptomatic. Symptoms can range from mild diarrhoea to abdominal cramps, vomiting and severe bloody diarrhoea, with ca 30% of cases requiring hospital admission. Overall, around 5% of cases (rising to 11% in children aged 1–4 years) develop haemolytic-uraemic syndrome (HUS), which is a severe multisystem condition that predominantly affects the kidneys and can be fatal [1].

In 2022, STEC was the third most commonly notified food-borne zoonotic pathogen in the European Union/European Economic Area (EU/EEA) countries [2]. Of the 29 EU/EEA countries reporting data for 2022, 25 reported 8,565 confirmed cases of STEC infection [3], with 71 food-borne STEC outbreaks reported by 14 countries [4].

The gastrointestinal tract of ruminants is the ecological niche of STEC, with cattle and sheep being the main animal reservoirs [5]. Transmission from animals to humans can occur via direct contact with colonised animals or their environment or by the consumption of food or water contaminated with the pathogen. Food items frequently associated with food-borne outbreaks of STEC O157 include raw or undercooked beef or lamb meat products, unpasteurised dairy products and fresh produce exposed to rainwater run-off, floodwater or irrigation water containing animal faeces [6].

Climate hazards have previously been described as having the potential to activate cascading risk pathways with a sequence of secondary, causally connected events [7]. For example, cascading risks associated with heavy precipitation followed by flooding of animal environments, may lead to contamination of crops and cause food-borne outbreaks of zoonotic diseases. Higher pathogen loads frequently detected in floodwater after rainstorms [8,9] and extreme weather events have been associated with outbreaks of gastrointestinal illness [10-12].

## Outbreak detection

In late August and early September 2022, the United Kingdom (UK) Health Security Agency (UKHSA) Gastrointestinal Bacteria Reference Unit (GBRU) reported a substantial increase in the submission of containment level 3 faecal samples and isolates that were presumptive for STEC. The number of presumptive STEC isolates received by the reference laboratory in the first 6 days of September 2022 was 245, compared with 259 isolates received during the whole of September 2019, the most recent pre-pandemic year for which data were comparable. Between 5 and 7 September, the number of confirmed STEC O157 cases was 73 compared with an average of 16 cases of STEC O157 reported each week in the previous 4 weeks. This, coupled with a substantial increase in the number of enhanced surveillance questionnaires (ESQs) for STEC cases suggested a surge in cases warranting further investigation. On 7 September 2022, a national level outbreak was declared and a multi-agency incident management team (IMT) established.

The IMT aimed to investigate the source of the outbreak by undertaking traditional epidemiological analyses and traceback investigations. A separate sub-group of the IMT explored a novel triangulation methodology using meteorological, flood risk, land use and land-classification and sheep holding density data, to explain the contamination and independently determine the potential source of the STEC outbreak.

## Methods

### Microbiology and genome sequencing

Isolates of presumptive STEC from hospital and local diagnostic laboratories across the UK were referred to the GBRU or the Scottish *E. coli* Reference Laboratory (SERL) for confirmation and genome sequencing. Briefly, Illumina (Illumina, San Diego, the United States (US)) reads were mapped to the STEC O157:H7 reference genome Sakai (GenBank accession: BA000007), and high-quality variants, single nucleotide polymorphism (SNPs), were identified [13]. Isolates with genome sequences that fall within a 5 SNP single linkage cluster are likely to be from the same source. FASTQ reads from sequences in this study from cases resident in England have been submitted to the National Centre for Biotechnology Information (NCBI; https://www.ncbi.nlm.nih.gov/) (BioProject No. PRJNA315192). Strain specific identifiers can be found in the Supplementary Table S1.

### Outbreak case definition

First whole genome sequencing (WGS) results became available on 9 September 2022, identifying two cases belonging to one specific 5-SNP cluster. By 11 September 2022, a further 19 cases belonging to the same cluster were notified and the investigation then focused on this specific genomic variant of STEC O157:H7. A confirmed case was defined as a case of STEC O157:H7 clonal complex (CC)11 notified since 1 January 2022 and resident in the UK, and (i) with a UKHSA SNP address: 24.223.1102.2049.4926.5294.% (t5:5294) or (ii) falling within t5.5294 after accounting for mobile genetic elements.

### Epidemiology

All confirmed cases of STEC O157 in England are requested to complete an ESQ by local health protection teams to capture demographics, clinical symptoms, environmental and food exposures [14]. These data are submitted to the UKHSA’s National Enhanced STEC Surveillance System (NESSS) and case line list data are generated. Descriptive epidemiological analyses were performed in Microsoft Excel and R (https://www.r-project.org/). This included a summary of available food exposure information, epidemiological curves, and age, sex and geographical distribution of the cases, travel reported and clinical severity of illness. Hypothesis generation interviews were undertaken 13–16 September 2022 using a targeted questionnaire proforma in telephone interviews.

A frequency-matched case–control study was undertaken across all four UK nations. Thirty-eight cases for the study were selected using stratified random sampling and interviewed via telephone between 28 September and 14 October 2022 using a bespoke questionnaire focusing on eating out, chicken, beef and salad products. A sample size calculation was performed with a ratio of four controls per case and no more than 30% of controls exposed. The calculations yielded a sample size of 38 cases and 152 unmatched controls, having at least 80% power to detect the difference if 50% of cases were exposed and no more than 30% of controls were exposed. A total of 190 controls (inflated to account for poor quality or non-response) were planned to be recruited but the final study recruited 41 cases and 206 controls. Controls were recruited via a market research panel and were frequency matched to cases on location of residence, age and sex and who did not have a history of diarrhoea and/or vomiting in the 7 days before completion of the control questionnaire.

Univariable logistic regression analysis of each exposure variable was undertaken and odds ratios (OR), p values and 95% confidence intervals (CI) were reported. Exposures with raised odds of illness and likelihood ratio test (LRT) p value < 0.2 from the univariable analysis were considered for inclusion in multivariable analysis (MVA) using Firth logistic regression and a forward stepwise approach. Two MVA models were constructed and undertaken in parallel for quality assurance purposes. Different statistical software packages were used, depending on user-preference: R version 4.2.1 and RStudio 2022.07.0 + 548 were used to produce the univariable analyses and model two, Stata version 17.0 (https://www.stata.com/) was used to produce model one. Models one and two were replicated in both R and Stata and yielded identical results.

Loyalty card data provided during the case interviews were used to inform requests for food purchase information from retailers for the time frame of interest. Data were also derived from the National Diet and Nutrition Survey (NDNS) [15] to estimate typical consumption of food items frequently reported by cases at the population level for comparative purposes.

Based on the signals derived from the epidemiological investigations above, traditional food chain investigations were employed. Concurrently and independent of the food chain investigations, a novel methodology using meteorological, flood risk, land use, land-classification and animal density data was explored.

### Food chain investigation

The Food Standards Agency (FSA) liaised with fresh produce trade bodies to establish possible emerging industry trends, processes and procedures at each stage of the supply chain, and the nature and detail of the risk assessments carried out pre- and post-harvest. To further understand operational procedures and practises at the fresh produce grower level, processing and packing stages, two site visits were undertaken to a fresh produce grower and a fresh produce grower and processor.

### Meteorological analysis (precipitation and temperature)

Land surface weather observation data were analysed for July and August 2022 from (i) the Meteorological (Met) Office Integrated Data Archive System (MIDAS) via the Environmental Public Health Surveillance System (EPHSS) [16] and (ii) the Environment Agency (EA) rainfall data available via the Hydrology Data Explorer [17]. The UK rainfall and mean daily maximum temperature anomaly maps were accessed under open government license from the Met Office for analyses [18]. Anomaly maps are based on gridded datasets (the HadUK-Grid dataset [19]), with the anomaly information referring to the 30-year averaging periods 1991–2020.

### Flood risk and land surface (land use and land classification) analysis

Maps indicating the annual likelihood of flooding from rivers and the sea in England were generated using data from the EA [20]. Agricultural land classification maps from Natural England were accessed and analysed to identify areas of land considered excellent for agricultural use (ALC005) [21]. Land use data were accessed and analysed to identify areas of land used for growing lettuce (Land Use Code: AC15) [22]. This was cross-examined with areas of interest identified through analysis of weather and animal density data.

### Animal population

Sheep holding density and sheep population density maps derived from The Sheep and Goat Inventory (December 2021–January 2022) produced by the Animal and Plant Health Agency (APHA) were accessed and analysed to assess the sheep population [23]. The data are based on a winter inventory which represents the adult breeding flocks and lambs kept for breeding and fattening. Most lambs are born in spring, and sheep numbers are ca 70% higher in the summer due to presence of lamb crop.

## Results

### Microbiology and genome sequencing

Phylogenetic analysis of the sequencing data showed that all isolates fell within a 5 SNP single linkage cluster (minimum = 0.71 SNPs; maximum = 11 SNPs; average < 1 SNP), providing strong evidence for a link to a common source. Seven isolates of STEC O157:H7 closely related to the outbreak strain (distance of 17 SNPs) were previously reported in 2019 (three cases), 2020 (two cases) and 2021 (two cases). The outbreak strain had both *stx1a* and *stx2c* genes and belonged to sub-lineage IIb, as presented in Supplementary Table 1.

### Descriptive epidemiology

**Figure 1:**
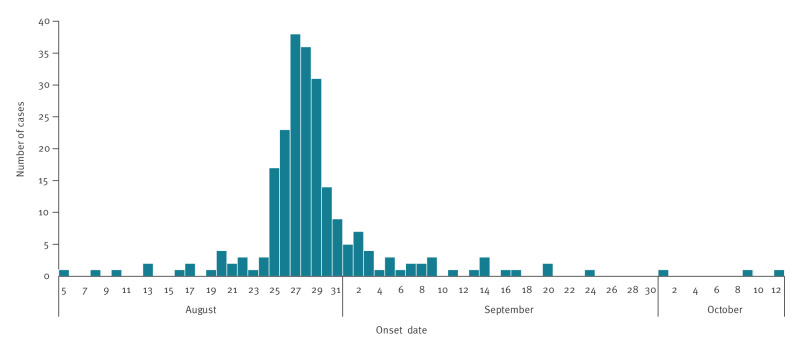
Temporal distribution of confirmed Shiga-toxin producing *Escherichia coli* (STEC) O157 t5.5294 cases, by symptom onset date, the United Kingdom, August–October 2022 (n = 231)

There were 259 cases, including seven secondary cases, linked to the outbreak, with symptom onset dates ranging from 5 August to 12 October 2022 (Figure 1). The index case had an onset date of 5 August, and the last notified primary case had an onset date of 20 September 2022. Cases were dispersed across all four nations of the UK. There was a higher proportion of female cases (n = 142; 55%) compared to male cases (n = 117; 45%). The age groups most affected were aged 20–29 years (n = 85; 33%) and 30–39 years (n = 52; 20%), followed by 10–19 years (n = 32; 12%), 40–49 years (n = 25; 10%), 50–59 years (n = 21; 8%), 0–9 years (n = 18; 7%), 60–69 years (n = 14; 5%), 70–79 years (n = 8; 3%) and ≥ 80 years (n = 4; 2%).

Symptom onset dates were not available for 28 confirmed cases.

Details on clinical symptoms and hospitalisation were available for 255 cases. The most commonly reported symptoms were diarrhoea (n = 234; 92%) and abdominal pain (n = 222; 87%), followed by blood in stools (n = 166; 65%), nausea (n = 134; 53%), vomiting (n = 82; 32%) and fever (n = 76; 30%). Seventy-seven (30%) confirmed cases attended hospital for their symptoms and 75 (29%) were admitted. None of the cases were diagnosed with HUS and no deaths were reported.

### Analytical epidemiology

Hypothesis generation interviews were undertaken with 19 cases with illness onsets between 10 and 29 August 2022. The most frequently reported exposures were having eaten out (19 cases), eating chicken (18 cases), any salad leaves or prepacked salad exposure (18 cases) and beef (12 cases). Data derived from the NDNS estimated typical consumption of these food types in the population over a 4-day period as: chicken (59%), salad leaves (65%) and beef (47%).

The univariable analysis of the frequency-matched case–control study showed positive association between exposure to chicken and illness (OR = 9.65; 95% CI: 2.48–87.0), with cases having an increased odds of exposure to eating chicken prepared away from the home. Consumption of any salad leaves (OR = 2.53; 95% CI: 1.15–6.29), lettuce (OR = 2.13; 95% CI: 1.02–4.87) and iceberg lettuce (OR = 3.06; 95% CI: 1.44–6.81) were all positively associated with illness; for any salad leaves and iceberg lettuce, an increased odds was seen in those that ate these items prepared away from the home.

Multivariable analysis (model 1) included composite level variables and indicated a strong association between illness and consumption of chicken away from the home at least some of the time (OR = 12.7; 95% CI: 2.24–72.2) (n = 247; 41 cases and 206 controls).

Multivariable analysis (model 2) included both composite and individual ingredient variables and indicated evidence of an association between illness and chicken pieces eaten at home or outside the home (OR = 5.1; 95% CI: 1.65–16.1). Consumption of iceberg lettuce, prepared at home or outside the home was also associated with illness (OR = 3.38; 95% CI: 1.35–9.23) (n = 214; 30 cases and 184 controls).

The authors make additional analysis outputs available in Supplementary Tables S2–S4.

### Food chain investigation

Based on the analysis of the case exposure information, chicken products and salad items were the main focus of the early food chain investigations. Since limited commonality was identified between types of products, purchase locations and supply chains, other than for lettuce, this food became the main focus of the food chain investigations.

Loyalty card data revealed no strong link to one type of salad leaf (lettuce, spinach, baby leaf spinach, cos romaine and other leaf types). The salad leaves were a mix of processed (sliced, chopped and bagged), as well as unprocessed, unwashed whole products. Supply chain information highlighted distribution of suspected salad products was to the UK and Ireland only.

For the period of interest (early August–early September), loyalty card data and supply chain investigations revealed Grower X, a lettuce grower, to be directly or indirectly linked via other growers, producers and suppliers, to all the food service establishments and/or retailers of interest (in total: 10 retailers, 7 processors, 25 suppliers, 3 food services, 4 manufacturers and 14 wholesalers).

The harvesting period for Grower X ended in October 2022. Grower X was visited by the competent authority after the harvest had ended (October–November 2022). A HACCP (hazard analysis and critical control points) plan was in place and legislative requirements were followed; there were no failures identified with regards to storage conditions or temperature control. Manure was not used as a fertiliser. Irrigation water systems were used to water the produce items during growth. Potable chlorinated water was used in processing, based on HACCP controls. Grower X reported that produce at the farm was exposed to standing water following heavy precipitation and local flooding. Routine testing during the harvest was undertaken by Grower X and shared with the competent authority during the investigation. Results revealed acceptable levels of indicator *E. coli* contamination of salad wash water. It had not been deemed necessary to remove crops from the supply chain at that time according to the HACCP plan and risk management protocols.

During the outbreak, no microbiological sampling of product was undertaken. Due to the complexity of the salad leaf supply chain, the short shelf life and the lack of specific batch information for product potentially consumed by the cases (particularly multi-leaf salad items), both the outbreak and the harvest period for Grower X had ended before Grower X was identified. Sampling of product and the environment was undertaken at later stages, after the investigation, to validate the efficacy of the food safety management system, findings were unremarkable.

### Meteorological analysis (precipitation and temperature)

We identified four rain gauges across the UK recording unusual daily totals of rain over 100 mm between 9:00 on 16 August and 09:00 on 17 August 2022. Based on early signals from the epidemiological investigations, we focused on the region known for its arable and horticultural farming (Region A) [24] and excluded the two locations outside of the region of interest which received 112.0 mm and 110.8 mm, respectively.

**Figure 2:**
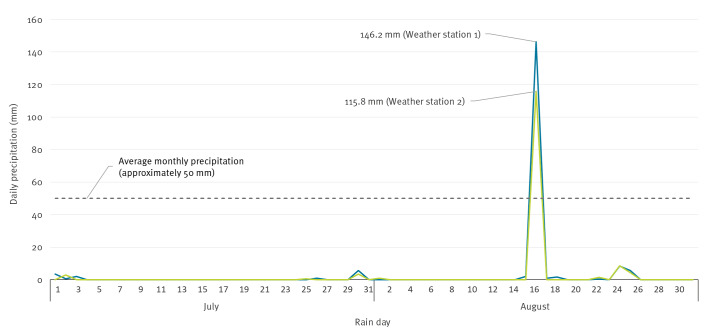
Daily precipitation recorded at Weather station 1 and Weather station 2 of Region A, the United Kingdom, July–August 2022

Data from MIDAS revealed one weather station (Weather station 1) in Region A had a total of 146.2 mm of rainfall 16–17 August 2022, with 115.4 mm of rainfall between 02:00 and 05:00 on 17 August 2022 (Figure 2).

Data analysed from the EA rain gauges also revealed unusual rainfall patterns at Weather station 2 in Region A which received a daily rainfall total of 115.8 mm on 16 August 2022. These two recordings are likely to have been caused by the same, slow-moving, weather system given their proximity (ca 20 km).

**Figure 3:**
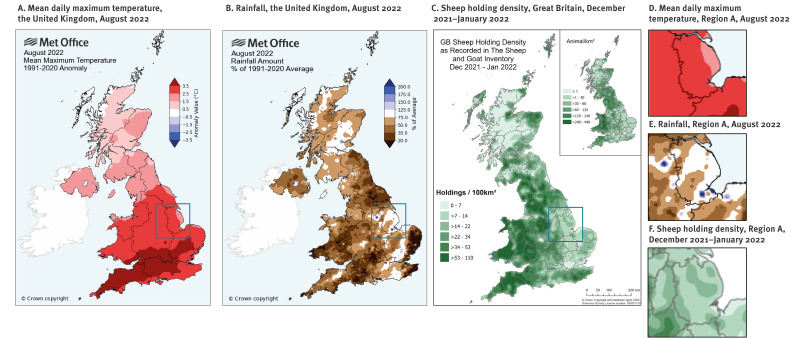
Maps presenting mean daily maximum temperature and rainfall (August 2022) and sheep holding density (December 2021–January 2022), the United Kingdom

Combined monthly accumulation of rainfall data and rainfall anomaly maps for August 2022 illustrate the two heavy, isolated rainfall events in Region A in an otherwise drought-like situation in the region, and across the UK (Figure 3A and Figure 3B).

A. Anomaly map illustrating mean daily maximum temperature of August 2022, expressed as the temperature deviation from the 1991–2020 averages.

B. Anomaly map illustrating rainfall amounts of August 2022, expressed as a percentage of 1991–2020 averages.

C. Sheep holding density map, Great Britain, December 2021–December 2022.

D. Anomaly map, daily maximum temperatures, Region A.

E. Anomaly map, rainfall, Region A.

F. Sheep holding density map, Region A.

Anomaly maps are available from the Met Office under open government licence. Sheep holding density maps are available from the Animal and Plant Health Agency under open government licence.

### Flood risk, land surface (land use, land classification and animal population) analysis

Animal holding density maps indicate there are sheep holdings (> 7 to 22 holdings/100km^2^) registered in the vicinity of the area of high rainfall. Animal population density maps also indicate that sheep are present in the area (albeit at lower levels on average compared to the rest of the Great Britain).

**Figure 4:**
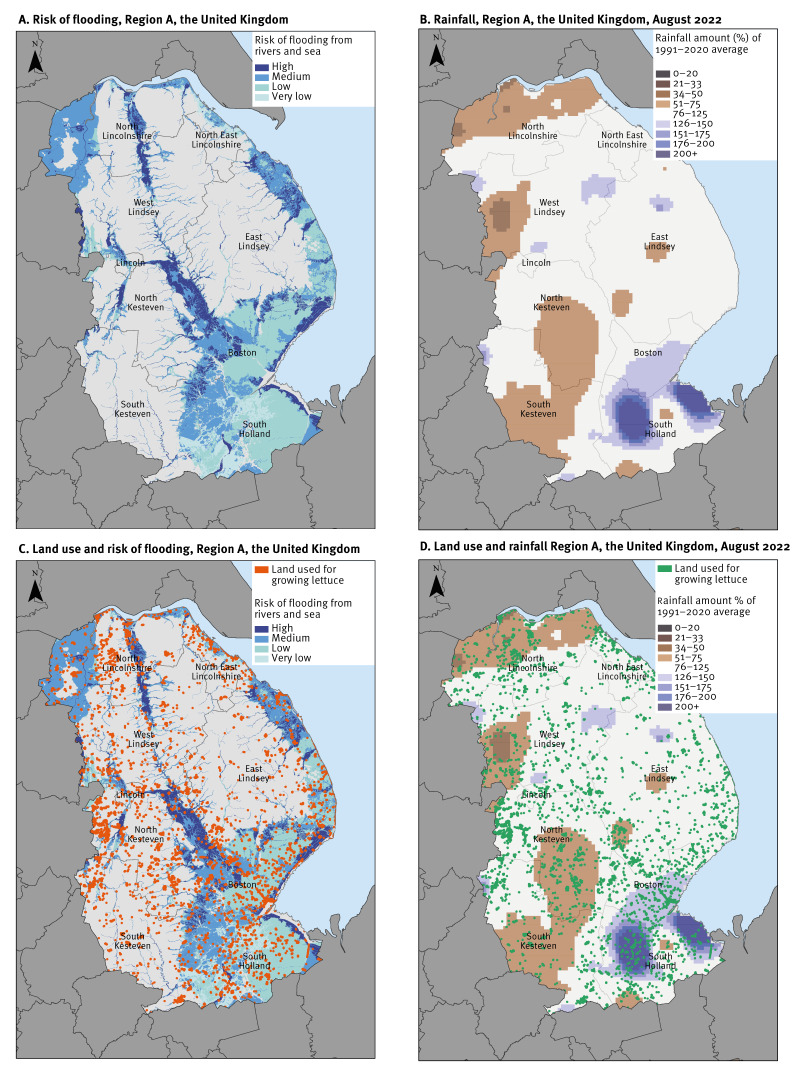
Maps presenting annual risk of flooding from rivers and sea, rainfall and land use, Region A, the United Kingdom

Geospatial analysis of flood risk data, land use data and land classification maps, animal density maps and subsequent Google Map searches for lettuce growers identified a plausible grower which was based in an area with:

- registered sheep holdings (Figure 3F),
- a higher risk of flooding (Figure 4A),
- unusual heavy precipitation events (Figure 4B),
- land being used for lettuce production (Figure 4C and 4D),
- a rating of ‘Excellent’ for agricultural use.

A. Flood risk map reproduced and adapted from Environment Agency flood risk data (2023).

B. Anomaly map illustrating rainfall in August 2022, expressed as a percentage of average rainfall amounts (1991-2020). Reproduced and adapted from Met Office Rainfall Anomaly Map.

C. Map illustrating areas used for lettuce production combined with Map A. Data from the Rural Payment Agency. Crop Map of England 2020.

D. Map B combined with areas used for lettuce production illustrated in Map C.

All maps contain Ordinance Survey data and National Statistics data. Crown copyright and database right 2023. All data available under open government licence.

The FSA subsequently confirmed that the grower identified independently from the meteorological, flood risk, land surface and animal population analysis was the same grower (Grower X) which was identified through the food chain investigation.

### Elucidation of the cascading risk pathway

**Figure 5:**
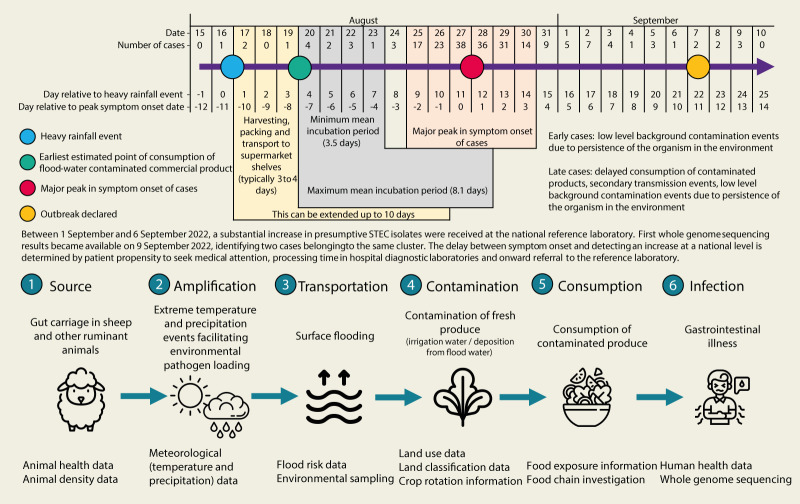
Timeline of events, cascading risk pathway and potential data sources, the United Kingdom, August–October 2022

A timeline of the events and a cascading risk pathway was developed based on the epidemiological, food chain and meteorological analysis (Figure 5).

## Outbreak response measures

Public health measures implemented as part of the outbreak included communications to diagnostic laboratories and clinicians to aid case-finding. Public health guidance was provided to cases in the form of an information leaflet which included general information about STEC and steps to take to prevent onward transmission of infection (personal hygiene measures, safe cooking and the need to stay away from work, school or nursery for at least 48 hours until after symptoms resolve).

Grower X was requested to review their HACCP plan to ensure hazards were identified fully, and that controls in place were suitable, in particular, contingency plans for extreme weather and flooding. A review of the efficacy of HACCP at growers' stages is ongoing; fresh produce growers and manufacturers have been requested to consider extreme weather and flooding as a hazard for gastrointestinal pathogens such as STEC and determine proportionate controls including pre- and post-harvesting checks, finished product testing and irrigation water used (sources, testing and corrective actions).

Due to the outbreak being over by the time the most likely implicated food supply chain had been identified, no product was recalled from the supply chain. Based on the evidence generated from the weather analysis, the FSA continues to actively engage with industry to inform risk assessment and risk management.

## Discussion

The outbreak described here was one of the largest outbreaks of STEC O157:H7 recorded in the UK since the first reported STEC outbreaks in the early 1980s. A combination of phylogenetic analyses, traditional epidemiological analyses, food chain investigations with a novel approach using data on upstream climate hazards led to the conclusion that domestically grown (UK) lettuce contaminated during an adverse flooding event, was the most plausible vehicle of infection. The analysis of climate-related factors independently confirmed the location of Grower X identified as the likely implicated producer of salad leaves in this outbreak.

The outbreak was detected following a rapid and unexpected increase in laboratory notifications of presumed STEC isolates, confirmed by genome sequencing to be STEC O157:H7 with a specific genetic profile defined at the 5-SNP level as the outbreak strain. Initial analysis suggested a nationally distributed food-borne source, as cases were geographically widely dispersed, with no other commonalities in basic descriptive epidemiological analyses identified such as attendance at large events or common travel destinations. Based on the descriptive and analytical and epidemiological study findings, both chicken and salad items were kept as an open line of enquiry, however, chicken consumed by cases were in a wide variety of forms and from different manufacturers which limited food chain investigations as there was no common food type or origin for the chicken products identified. It was considered by the IMT that cross-contamination could have played a role. As investigations continued, the evidence for salad items became more conclusive and investigations therefore focused on salad items as the likely vehicle. In recent times, other outbreaks of STEC O157:H7 involving over 200 cases have been caused by handling raw vegetables [25], imported herbs [26] and imported salad leaves [27].

A limitation of this study was that no microbiological sampling was done from known animal reservoirs, product or environment. We cannot definitively conclude that this outbreak was zoonotic in origin, however, zoonotic transmission from a domestic ovine reservoir is the most plausible explanation. The outbreak strain had *stx1a* and *stx2c* genes and belonged to sub-lineage IIb, which has been endemic in the UK sheep population since the 1980s [28], and sheep populations are known to be in proximity to Grower X. Analysis of the phylogenetic context of the outbreak strain indicated a domestic, ovine reservoir [29]. Small clusters of cases infected with sub-lineage IIb have previously been linked to lamb meat products including lamb mince and sausages made from lamb mince and pre-packed mixed salad leaves [30]. Furthermore, contamination of fresh produce due to persistence of the organism in the environment has also been linked to recurrent outbreaks of the same strain of STEC O157, previously [31,32].

Food chain enquiries by the FSA confirmed domestically grown fresh produce was more likely causing the outbreak than an imported food item. Although there was no microbiological evidence from food or environmental samples, and despite the highly complex nature of the food chain, investigations by the FSA implicated one particular grower of interest (Grower X) which was found to be directly or indirectly linked to all the food service establishments or retailers of interest. The annual harvest of Grower X’s crop had finished before Grower X was identified, and after the outbreak had ended, making it difficult to verify the conclusions of the outbreak investigation through microbiological sampling of product. There was evidence of low-level indicator *E. coli* contamination of salad wash water. Inspection of the farm did not identify any evidence of poor practice. Adverse weather conditions in the UK involving unusually high temperatures followed by heavy rain causing a flooding event, could have amplified contamination and spread of the pathogen. These conditions were reported by Grower X during the period of interest in the outbreak investigation. A recent study considering exposure and case data of 23 years found the effects of storm-related rainfall have also been associated with a 48% increase in STEC infection 1 week after the storm [33].

It is well established that outbreaks of gastrointestinal disease caused by contaminated fresh produce can be difficult to resolve [6]. Due to the inherent delay between onset of disease and outbreak detection and poor patient recall of salad garnishes and side dishes, by the time a vehicle has been identified, the contaminated batch is usually unavailable for microbiological testing due to the short shelf life of the product. Lettuce and salad leaves entering the food chain in the UK are almost exclusively domestically produced in the UK during the summer months and almost exclusively imported from other European countries during the autumn and winter months [34], and the fresh produce supply chains are complex. In this outbreak, the food chain involved multiple growers, each owning multiple farms and all interacting with multiple processors, suppliers, wholesalers and retailers. Furthermore, as was the case in this outbreak, salad leaves are commonly sold as a mixed leaf, with components derived from multiple different producers, thus adding further complexity. These complex just-in-time supply chains result in produce from a single farm ultimately being supplied to many different retailers and food outlets. Unravelling the supply chain and working backwards to identify the outbreak source is challenging and time consuming.

A sequence of adverse weather events in July and August 2022 caused prolonged periods of extremely dry weather causing increased dust in crop areas followed by excess rain, which could have caused soil wash, standing water and flooding. Average annual precipitation in the UK typically ranges from ca 800 mm to 1,400 mm. Typical annual rainfall amounts in the region of interest are around 600 mm [35]. Analysis of meteorological data revealed over 2 months of rainfall recorded in an otherwise drought-like situation in Region A where Grower X was located. For comparison, the record for highest 3-hour accumulation available from the Met Office is 178 mm for 7 October 1960 and for 1-hour is 92 mm on 12 July 1901.

The adverse weather conditions occurred within 2 weeks of the major peak of case symptom onset dates. This was consistent with the expected time taken for fresh lettuce produce to make its way from farm to fork added to the known incubation period following exposure to STEC. Typically, lettuce is sold in supermarkets 3 to 4 days post harvesting and packing, which can be extended to 10 days [36]. A systemic review of 28 outbreak investigation studies found the mean incubation period to range from 3.5 to 8.1 days [37]. It is plausible, therefore, that the unusual precipitation events on dry, impermeable soil led to localised flooding, facilitating the amplification and transport of food-borne pathogens, crop contamination and the subsequent STEC O157:H7 outbreak.

Despite the peak of case symptom onset dates being within 2 weeks of the adverse weather conditions, a small number of cases pre-dated the weather event (early cases) and a small number, predominantly secondary case reports in late September and October 2022 (late cases), after the outbreak peak in late August and early September 2022. We hypothesise that both early and late primary cases, as well as the cases notified in previous years, could be explained by low-level environmental contamination due to persistence of the organism in the environment.

**Figure 6:**
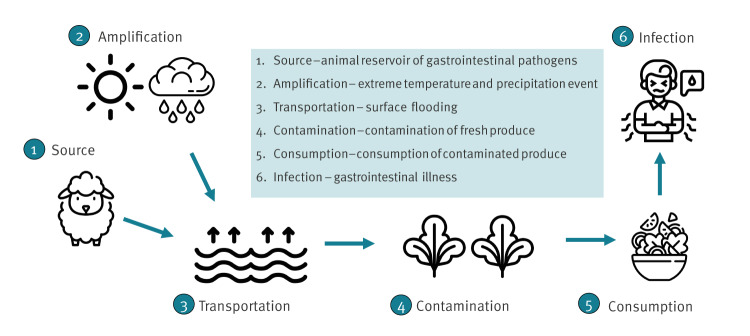
Hypothesised cascading risk pathway of weather events on Shiga-toxin producing *Escherichia coli* (STEC) outbreaks associated with fresh produce

Contamination of crops could have occurred via three cascading weather-related routes including (Figure 6):

- heavy rainfall washing pathogens from animal pastures into fields with crop causing direct contamination through surface flooding,
- localised flooding contaminating water supply subsequently used to irrigate crops,
- cross-contamination of fresh produce washing and handling arising due to greater contamination of crops as a result of increased exposure to contaminated dust, floodwater and/or soil.

We present a novel approach with the collation and analysis of additional data available on weather, flood, animal and land use data, integrated with traditional approaches as a useful addition to evidence generation in certain food-borne disease outbreaks. This demonstrates that if siloed datasets are analysed through the lens of One Health, benefits could be realised for both food security and human health security. We recommend future similar analyses plan and explore the use sampling of the environment in proximity to animal production premises and incorporation of weather and land use data for a true One Health approach.

Analyses such as this could be automated (through machine learning and artificial intelligence) and used either predictively or in real time to:

- form the basis of a novel One Health early warning surveillance system for high-risk amplification events in certain food- and waterborne pathogen outbreaks,
- inform risk mitigation strategies and food safety measures such as targeted microbiological testing of product,
- support evidence generation in emerging outbreaks to help identify the source early in an outbreak.

Policy plays a key role in the prevention of outbreaks such as this one. The FSA has produced guidelines for growers to reduce the risks of microbiological contamination of ready-to-eat crops from farm manures [38]. Although there are no specific UK or EU regulations for flooding, the FSA advises that “fruit or vegetables to be eaten raw should not be harvested for at least 6 months after the floodwater has receded” [39]. In the EU, the Commission notice on guidance document on addressing microbiological risks in fresh fruits and vegetables at primary production through good hygiene states: “Fresh fruit and vegetables for which the edible part has come into contact with floodwaters close to harvest (less than 2 weeks) should not be consumed as raw product. If the flooding event takes place more than 2 weeks before harvest or if these products are processed, a case-by-case (site-specific) risk assessment should be performed” [40]. Our analysis provides evidence to support such recommendations.

Our approach has the potential to be developed for other infectious gastrointestinal pathogens considered to be climate-sensitive, including *Salmonella* and *Campylobacte*r, *Cryptosporidium* and *Giardia* [41]. The world is already experiencing changes in average temperature, shifts in the seasons and an increasing frequency of extreme weather events. The frequency of flooding events is only expected to increase with atmospheric warming associated with climate change [42,43]. Climate-informed surveillance and response systems for key risks such as extreme weather events and infectious disease burden will be critical in supporting the World Health Organization’s drive for climate adaptation and resilience in health determining sectors including food and water [44].

Surveillance and investigation approaches which go further upstream, require the development and elucidation of cascading risk pathways, followed by the interrogation of relevant and novel data sources. Many of the relevant data sources (meteorological and land use data) are often already available. Multi-disciplinary, multi-agency, cross-sectoral collaboration on a One Health basis, open data-sharing and engagement with industry will be essential to realise the potential public health benefits.

## Conclusion

Fresh produce contamination events are typically transient, and the resulting outbreaks often end before intervention measures can be implemented. Nevertheless, there is increasing evidence to show that outbreaks linked to fresh produce and caused by the same whole genome sequenced defined strain of bacteria can re-occur year after year. Therefore, it is important to resolve the root cause of such outbreaks, make recommendations and implement preventative and control measures, through evidence-based policy approaches to avoid recurrence. It is challenging to identify proportionate actions that could be taken to reduce the risk of contaminated fresh produce being sold to the public following adverse weather events. In the future, proactive utilisation of real-time weather monitoring data could be used by risk assessors and risk managers to identify farms experiencing adverse weather events that could result in amplification of risk, to enhance pre- and post-harvest risk mitigation measures (such as microbiological testing). Therefore, we recommend more routine use of weather, animal and land data incorporated into outbreak investigation approaches to correlate heavy rainfall patterns and other unusual weather events with outbreaks and incidence of food-borne gastrointestinal disease, to inform the evidence base, and to better understand the impact of climate change on public health.

## Supplementary Data

349000 Supplementary Material
