# Supplementary material for: An outbreak of Shiga toxin-producing Escherichia coli (STEC) O157:H7 associated with contaminated lettuce and the cascading risks from climate change, the United Kingdom, August to September 2022
Source: Euro Surveill. 2024 Sep 5;29(36):2400161. doi: 10.2807/1560-7917.ES.2024.29.36.2400161 (PMC11378517; doi:10.2807/1560-7917.ES.2024.29.36.2400161)
Supplement: Supplementary Material [file 24-00161_CUNNINGHAM_Supplement.pdf]

Supplementary Table S1. Table detailing the NCBI FASTQ SRA accessions for finalised assemblies from cases resident in England.

	External ID	FASTQ SRA accession no.	Year	Organism	Serotype	ST	CC	Lineage	EAE	Stx1	Stx2	Stx Subtype
1	813445	SRR10228115	2019	Escherichia coli	O157:H7	11	CC11	IIb	+	+	+	stx1a,stx2c
2	813443	SRR10228118	2019	Escherichia coli	O157:H7	11	CC11	IIb	+	+	+	stx1a,stx2c
3	953817	SRR12037674	2020	Escherichia coli	O157:H7	11	CC11	IIb	+	+	+	stx1a,stx2c
4	988797	SRR12676728	2020	Escherichia coli	O157:H7	11	CC11	IIb	+	+	+	stx1a,stx2c
5	991428	SRR12714035	2020	Escherichia coli	O157:H7	11	CC11	IIb	+	+	+	stx1a,stx2c
6	1507349	SRR16598718	2021	Escherichia coli	O157:H7	11	CC11	IIb	+	+	+	stx1a,stx2c
7	1502228	SRR16612615	2021	Escherichia coli	O157:H7	11	CC11	IIb	+	+	+	stx1a,stx2c
8	1698389	SRR21488028	2022	Escherichia coli	O157:H7	11	CC11	IIb	+	+	+	stx1a,stx2c
9	1699281	SRR21504750	2022	Escherichia coli	O157:H7	11	CC11	IIb	+	+	+	stx1a,stx2c
10	1699275	SRR21504751	2022	Escherichia coli	O157:H7	11	CC11	IIb	+	+	+	stx1a,stx2c
11	1699316	SRR21504756	2022	Escherichia coli	O157:H7	11	CC11	IIb	+	+	+	stx1a,stx2c
12	1699286	SRR21504760	2022	Escherichia coli	O157:H7	11	CC11	IIb	+	+	+	stx1a,stx2c
13	1699403	SRR21504763	2022	Escherichia coli	O157:H7	11	CC11	IIb	+	+	+	stx1a,stx2c
14	1699273	SRR21504765	2022	Escherichia coli	O157:H7	11	CC11	IIb	+	+	+	stx1a,stx2c
15	1699296	SRR21504767	2022	Escherichia coli	O157:H7	11	CC11	IIb	+	+	+	stx1a,stx2c
16	1699276	SRR21504770	2022	Escherichia coli	O157:H7	11	CC11	IIb	+	+	+	stx1a,stx2c
17	1699268	SRR21504773	2022	Escherichia coli	O157:H7	11	CC11	IIb	+	+	+	stx1a,stx2c
18	1699256	SRR21504778	2022	Escherichia coli	O157:H7	11	CC11	IIb	+	+	+	stx1a,stx2c
19	1699317	SRR21504779	2022	Escherichia coli	O157:H7	11	CC11	IIb	+	+	+	stx1a,stx2c
20	1699283	SRR21504782	2022	Escherichia coli	O157:H7	11	CC11	IIb	+	+	+	stx1a,stx2c
21	1699264	SRR21504783	2022	Escherichia coli	O157:H7	11	CC11	IIb	+	+	+	stx1a,stx2c
22	1699298	SRR21504790	2022	Escherichia coli	O157:H7	11	CC11	IIb	+	+	+	stx1a,stx2c
23	1699253	SRR21504792	2022	Escherichia coli	O157:H7	11	CC11	IIb	+	+	+	stx1a,stx2c
24	1699270	SRR21504794	2022	Escherichia coli	O157:H7	11	CC11	IIb	+	+	+	stx1a,stx2c
25	1699312	SRR21504802	2022	Escherichia coli	O157:H7	11	CC11	IIb	+	+	+	stx1a,stx2c
26	1699297	SRR21504806	2022	Escherichia coli	O157:H7	11	CC11	IIb	+	+	+	stx1a,stx2c
27	1700619	SRR21520302	2022	Escherichia coli	O157:H7	11	CC11	IIb	+	+	+	stx1a,stx2c
28	1700654	SRR21520308	2022	Escherichia coli	O157:H7	11	CC11	IIb	+	+	+	stx1a,stx2c
29	1700607	SRR21520310	2022	Escherichia coli	O157:H7	11	CC11	IIb	+	+	+	stx1a,stx2c
30	1700591	SRR21520312	2022	Escherichia coli	O157:H7	11	CC11	IIb	+	+	+	stx1a,stx2c
31	1700655	SRR21520313	2022	Escherichia coli	O157:H7	11	CC11	IIb	+	+	+	stx1a,stx2c
32	1700569	SRR21520314	2022	Escherichia coli	O157:H7	11	CC11	IIb	+	+	+	stx1a,stx2c
33	1700633	SRR21520315	2022	Escherichia coli	O157:H7	11	CC11	IIb	+	+	+	stx1a,stx2c
34	1700620	SRR21520317	2022	Escherichia coli	O157:H7	11	CC11	IIb	+	+	+	stx1a,stx2c
35	1700626	SRR21520318	2022	Escherichia coli	O157:H7	11	CC11	IIb	+	+	+	stx1a,stx2c
36	1700551	SRR21520319	2022	Escherichia coli	O157:H7	11	CC11	IIb	+	+	+	stx1a,stx2c
37	1700623	SRR21520322	2022	Escherichia coli	O157:H7	11	CC11	IIb	+	+	+	stx1a,stx2c

This supplementary material is hosted by Eurosurveillance as supporting information alongside the article '*An outbreak of Shiga toxin-producing Escherichia coli (STEC) O157:H7 associated with contaminated lettuce and the cascading risks from climate change, the United Kingdom, August to September 2022'* on behalf of the authors, who remain responsible for the accuracy and appropriateness of the content. The same standards for ethics, copyright, attributions and permissions as for the article apply. Supplements are not edited by Eurosurveillance and the journal is not responsible for the maintenance of any links or email addresses provided therein

38	1700516	SRR21520323	2022	Escherichia coli	O157:H7	11	CC11	IIb	+	+	+	stx1a,stx2c
39	1700643	SRR21520425	2022	Escherichia coli	O157:H7	11	CC11	IIb	+	+	+	stx1a,stx2c
40	1700524	SRR21520426	2022	Escherichia coli	O157:H7	11	CC11	IIb	+	+	+	stx1a,stx2c
41	1700637	SRR21520427	2022	Escherichia coli	O157:H7	11	CC11	IIb	+	+	+	stx1a,stx2c
42	1700577	SRR21520429	2022	Escherichia coli	O157:H7	11	CC11	IIb	+	+	+	stx1a,stx2c
43	1700614	SRR21520432	2022	Escherichia coli	O157:H7	11	CC11	IIb	+	+	+	stx1a,stx2c
44	1700647	SRR21520435	2022	Escherichia coli	O157:H7	11	CC11	IIb	+	+	+	stx1a,stx2c
45	1700608	SRR21520436	2022	Escherichia coli	O157:H7	11	CC11	IIb	+	+	+	stx1a,stx2c
46	1700561	SRR21520437	2022	Escherichia coli	O157:H7	11	CC11	IIb	+	+	+	stx1a,stx2c
47	1700650	SRR21520441	2022	Escherichia coli	O157:H7	11	CC11	IIb	+	+	+	stx1a,stx2c
48	1700635	SRR21520445	2022	Escherichia coli	O157:H7	11	CC11	IIb	+	+	+	stx1a,stx2c
49	1700612	SRR21520446	2022	Escherichia coli	O157:H7	11	CC11	IIb	+	+	+	stx1a,stx2c
50	1700568	SRR21520449	2022	Escherichia coli	O157:H7	11	CC11	IIb	+	+	+	stx1a,stx2c
51	1700547	SRR21520450	2022	Escherichia coli	O157:H7	11	CC11	IIb	+	+	+	stx1a,stx2c
52	1700581	SRR21520451	2022	Escherichia coli	O157:H7	11	CC11	IIb	+	+	+	stx1a,stx2c
53	1700657	SRR21520453	2022	Escherichia coli	O157:H7	11	CC11	IIb	+	+	+	stx1a,stx2c
54	1700600	SRR21520454	2022	Escherichia coli	O157:H7	11	CC11	IIb	+	+	+	stx1a,stx2c
55	1700594	SRR21520455	2022	Escherichia coli	O157:H7	11	CC11	IIb	+	+	+	stx1a,stx2c
56	1700641	SRR21520456	2022	Escherichia coli	O157:H7	11	CC11	IIb	+	+	+	stx1a,stx2c
57	1700527	SRR21520457	2022	Escherichia coli	O157:H7	11	CC11	IIb	+	+	+	stx1a,stx2c
58	1700645	SRR21520458	2022	Escherichia coli	O157:H7	11	CC11	IIb	+	+	+	stx1a,stx2c
59	1700598	SRR21520462	2022	Escherichia coli	O157:H7	11	CC11	IIb	+	+	+	stx1a,stx2c
60	1700555	SRR21520522	2022	Escherichia coli	O157:H7	11	CC11	IIb	+	+	+	stx1a,stx2c
61	1700599	SRR21520524	2022	Escherichia coli	O157:H7	11	CC11	IIb	+	+	+	stx1a,stx2c
62	1700558	SRR21520526	2022	Escherichia coli	O157:H7	11	CC11	IIb	+	+	+	stx1a,stx2c
63	1700586	SRR21520527	2022	Escherichia coli	O157:H7	11	CC11	IIb	+	+	+	stx1a,stx2c
64	1700597	SRR21520528	2022	Escherichia coli	O157:H7	11	CC11	IIb	+	+	+	stx1a,stx2c
65	1700653	SRR21520529	2022	Escherichia coli	O157:H7	11	CC11	IIb	+	+	+	stx1a,stx2c
66	1700634	SRR21520533	2022	Escherichia coli	O157:H7	11	CC11	IIb	+	+	+	stx1a,stx2c
67	1700652	SRR21520537	2022	Escherichia coli	O157:H7	11	CC11	IIb	+	+	+	stx1a,stx2c
68	1700631	SRR21520538	2022	Escherichia coli	O157:H7	11	CC11	IIb	+	+	+	stx1a,stx2c
69	1700639	SRR21520539	2022	Escherichia coli	O157:H7	11	CC11	IIb	+	+	+	stx1a,stx2c
70	1702414	SRR21524998	2022	Escherichia coli	O157:H7	11	CC11	IIb	+	+	+	stx1a,stx2c
71	1702348	SRR21525001	2022	Escherichia coli	O157:H7	11	CC11	IIb	+	+	+	stx1a,stx2c
72	1702304	SRR21525005	2022	Escherichia coli	O157:H7	11	CC11	IIb	+	+	+	stx1a,stx2c
73	1702454	SRR21525007	2022	Escherichia coli	O157:H7	11	CC11	IIb	+	+	+	stx1a,stx2c
74	1702353	SRR21525008	2022	Escherichia coli	O157:H7	11	CC11	IIb	+	+	+	stx1a,stx2c
75	1702408	SRR21525010	2022	Escherichia coli	O157:H7	11	CC11	IIb	+	+	+	stx1a,stx2c
76	1702447	SRR21525012	2022	Escherichia coli	O157:H7	11	CC11	IIb	+	+	+	stx1a,stx2c
77	1702440	SRR21525013	2022	Escherichia coli	O157:H7	11	CC11	IIb	+	+	+	stx1a,stx2c

This supplementary material is hosted by Eurosurveillance as supporting information alongside the article '*An outbreak of Shiga toxin-producing Escherichia coli (STEC) O157:H7 associated with contaminated lettuce and the cascading risks from climate change, the United Kingdom, August to September 2022'* on behalf of the authors, who remain responsible for the accuracy and appropriateness of the content. The same standards for ethics, copyright, attributions and permissions as for the article apply. Supplements are not edited by Eurosurveillance and the journal is not responsible for the maintenance of any links or email addresses provided therein

78	1702360	SRR21525019	2022	Escherichia coli	O157:H7	11	CC11	IIb	+	+	+	stx1a,stx2c
79	1702312	SRR21525022	2022	Escherichia coli	O157:H7	11	CC11	IIb	+	+	+	stx1a,stx2c
80	1702439	SRR21525025	2022	Escherichia coli	O157:H7	11	CC11	IIb	+	+	+	stx1a,stx2c
81	1702405	SRR21525026	2022	Escherichia coli	O157:H7	11	CC11	IIb	+	+	+	stx1a,stx2c
82	1700628	SRR21525027	2022	Escherichia coli	O157:H7	11	CC11	IIb	+	+	+	stx1a,stx2c
83	1702413	SRR21525032	2022	Escherichia coli	O157:H7	11	CC11	IIb	+	+	+	stx1a,stx2c
84	1702305	SRR21525033	2022	Escherichia coli	O157:H7	11	CC11	IIb	+	+	+	stx1a,stx2c
85	1702337	SRR21525035	2022	Escherichia coli	O157:H7	11	CC11	IIb	+	+	+	stx1a,stx2c
86	1702456	SRR21525042	2022	Escherichia coli	O157:H7	11	CC11	IIb	+	+	+	stx1a,stx2c
87	1702388	SRR21525044	2022	Escherichia coli	O157:H7	11	CC11	IIb	+	+	+	stx1a,stx2c
88	1702412	SRR21525047	2022	Escherichia coli	O157:H7	11	CC11	IIb	+	+	+	stx1a,stx2c
89	1702309	SRR21525048	2022	Escherichia coli	O157:H7	11	CC11	IIb	+	+	+	stx1a,stx2c
90	1702442	SRR21525052	2022	Escherichia coli	O157:H7	11	CC11	IIb	+	+	+	stx1a,stx2c
91	1702336	SRR21525054	2022	Escherichia coli	O157:H7	11	CC11	IIb	+	+	+	stx1a,stx2c
92	1702358	SRR21525056	2022	Escherichia coli	O157:H7	11	CC11	IIb	+	+	+	stx1a,stx2c
93	1702316	SRR21525057	2022	Escherichia coli	O157:H7	11	CC11	IIb	+	+	+	stx1a,stx2c
94	1702335	SRR21525060	2022	Escherichia coli	O157:H7	11	CC11	IIb	+	+	+	stx1a,stx2c
95	1702387	SRR21525061	2022	Escherichia coli	O157:H7	11	CC11	IIb	+	+	+	stx1a,stx2c
96	1702451	SRR21525065	2022	Escherichia coli	O157:H7	11	CC11	IIb	+	+	+	stx1a,stx2c
97	1702310	SRR21525078	2022	Escherichia coli	O157:H7	11	CC11	IIb	+	+	+	stx1a,stx2c
98	1702299	SRR21525079	2022	Escherichia coli	O157:H7	11	CC11	IIb	+	+	+	stx1a,stx2c
99	1702350	SRR21525081	2022	Escherichia coli	O157:H7	11	CC11	IIb	+	+	+	stx1a,stx2c
100	1702401	SRR21525082	2022	Escherichia coli	O157:H7	11	CC11	IIb	+	+	+	stx1a,stx2c
101	1702297	SRR21525084	2022	Escherichia coli	O157:H7	11	CC11	IIb	+	+	+	stx1a,stx2c
102	1702303	SRR21525086	2022	Escherichia coli	O157:H7	11	CC11	IIb	+	+	+	stx1a,stx2c
103	1702331	SRR21525087	2022	Escherichia coli	O157:H7	11	CC11	IIb	+	+	+	stx1a,stx2c
104	1702342	SRR21525092	2022	Escherichia coli	O157:H7	11	CC11	IIb	+	+	+	stx1a,stx2c
105	1702379	SRR21525093	2022	Escherichia coli	O157:H7	11	CC11	IIb	+	+	+	stx1a,stx2c
106	1702421	SRR21525096	2022	Escherichia coli	O157:H7	11	CC11	IIb	+	+	+	stx1a,stx2c
107	1702455	SRR21525097	2022	Escherichia coli	O157:H7	11	CC11	IIb	+	+	+	stx1a,stx2c
108	1702355	SRR21525099	2022	Escherichia coli	O157:H7	11	CC11	IIb	+	+	+	stx1a,stx2c
109	1702306	SRR21525103	2022	Escherichia coli	O157:H7	11	CC11	IIb	+	+	+	stx1a,stx2c
110	1702343	SRR21525106	2022	Escherichia coli	O157:H7	11	CC11	IIb	+	+	+	stx1a,stx2c
111	1702311	SRR21525108	2022	Escherichia coli	O157:H7	11	CC11	IIb	+	+	+	stx1a,stx2c
112	1700648	SRR21525109	2022	Escherichia coli	O157:H7	11	CC11	IIb	+	+	+	stx1a,stx2c
113	1702356	SRR21525110	2022	Escherichia coli	O157:H7	11	CC11	IIb	+	+	+	stx1a,stx2c
114	1702325	SRR21525113	2022	Escherichia coli	O157:H7	11	CC11	IIb	+	+	+	stx1a,stx2c
115	1702352	SRR21525141	2022	Escherichia coli	O157:H7	11	CC11	IIb	+	+	+	stx1a,stx2c
116	1702301	SRR21525144	2022	Escherichia coli	O157:H7	11	CC11	IIb	+	+	+	stx1a,stx2c
117	1702332	SRR21525147	2022	Escherichia coli	O157:H7	11	CC11	IIb	+	+	+	stx1a,stx2c

This supplementary material is hosted by Eurosurveillance as supporting information alongside the article '*An outbreak of Shiga toxin-producing Escherichia coli (STEC) O157:H7 associated with contaminated lettuce and the cascading risks from climate change, the United Kingdom, August to September 2022'* on behalf of the authors, who remain responsible for the accuracy and appropriateness of the content. The same standards for ethics, copyright, attributions and permissions as for the article apply. Supplements are not edited by Eurosurveillance and the journal is not responsible for the maintenance of any links or email addresses provided therein

118	1702428	SRR21525155	2022	Escherichia coli	O157:H7	11	CC11	IIb	+	+	+	stx1a,stx2c
119	1702448	SRR21533447	2022	Escherichia coli	O157:H7	11	CC11	IIb	+	+	+	stx1a,stx2c
120	1702427	SRR21533462	2022	Escherichia coli	O157:H7	11	CC11	IIb	+	+	+	stx1a,stx2c
121	1702398	SRR21533464	2022	Escherichia coli	O157:H7	11	CC11	IIb	+	+	+	stx1a,stx2c
122	1702429	SRR21533466	2022	Escherichia coli	O157:H7	11	CC11	IIb	+	+	+	stx1a,stx2c
123	1702346	SRR21533505	2022	Escherichia coli	O157:H7	11	CC11	IIb	+	+	+	stx1a,stx2c
124	1702313	SRR21533644	2022	Escherichia coli	O157:H7	11	CC11	IIb	+	+	+	stx1a,stx2c
125	1704309	SRR21607542	2022	Escherichia coli	O157:H7	11	CC11	IIb	+	+	+	stx1a,stx2c
126	1703573	SRR21607548	2022	Escherichia coli	O157:H7	11	CC11	IIb	+	+	+	stx1a,stx2c
127	1704302	SRR21607552	2022	Escherichia coli	O157:H7	11	CC11	IIb	+	+	+	stx1a,stx2c
128	1704291	SRR21607556	2022	Escherichia coli	O157:H7	11	CC11	IIb	+	+	+	stx1a,stx2c
129	1704299	SRR21607560	2022	Escherichia coli	O157:H7	11	CC11	IIb	+	+	+	stx1a,stx2c
130	1704284	SRR21607565	2022	Escherichia coli	O157:H7	11	CC11	IIb	+	+	+	stx1a,stx2c
131	1704312	SRR21607624	2022	Escherichia coli	O157:H7	11	CC11	IIb	+	+	+	stx1a,stx2c
132	1704303	SRR21607682	2022	Escherichia coli	O157:H7	11	CC11	IIb	+	+	+	stx1a,stx2c
133	1704351	SRR21607684	2022	Escherichia coli	O157:H7	11	CC11	IIb	+	+	+	stx1a,stx2c
134	1703552	SRR21607695	2022	Escherichia coli	O157:H7	11	CC11	IIb	+	+	+	stx1a,stx2c
135	1704300	SRR21607699	2022	Escherichia coli	O157:H7	11	CC11	IIb	+	+	+	stx1a,stx2c
136	1704343	SRR21607700	2022	Escherichia coli	O157:H7	11	CC11	IIb	+	+	+	stx1a,stx2c
137	1703559	SRR21607702	2022	Escherichia coli	O157:H7	11	CC11	IIb	+	+	+	stx1a,stx2c
138	1704325	SRR21607706	2022	Escherichia coli	O157:H7	11	CC11	IIb	+	+	+	stx1a,stx2c
139	1704354	SRR21607710	2022	Escherichia coli	O157:H7	11	CC11	IIb	+	+	+	stx1a,stx2c
140	1704347	SRR21607711	2022	Escherichia coli	O157:H7	11	CC11	IIb	+	+	+	stx1a,stx2c
141	1704336	SRR21607717	2022	Escherichia coli	O157:H7	11	CC11	IIb	+	+	+	stx1a,stx2c
142	1704285	SRR21607721	2022	Escherichia coli	O157:H7	11	CC11	IIb	+	+	+	stx1a,stx2c
143	1703558	SRR21607722	2022	Escherichia coli	O157:H7	11	CC11	IIb	+	+	+	stx1a,stx2c
144	1703535	SRR21607723	2022	Escherichia coli	O157:H7	11	CC11	IIb	+	+	+	stx1a,stx2c
145	1704356	SRR21607731	2022	Escherichia coli	O157:H7	11	CC11	IIb	+	+	+	stx1a,stx2c
146	1704326	SRR21607980	2022	Escherichia coli	O157:H7	11	CC11	IIb	+	+	+	stx1a,stx2c
147	1704348	SRR21616140	2022	Escherichia coli	O157:H7	11	CC11	IIb	+	+	+	stx1a,stx2c
148	1705768	SRR21618942	2022	Escherichia coli	O157:H7	11	CC11	IIb	+	+	+	stx1a,stx2c
149	1705693	SRR21618947	2022	Escherichia coli	O157:H7	11	CC11	IIb	+	+	+	stx1a,stx2c
150	1705697	SRR21618954	2022	Escherichia coli	O157:H7	11	CC11	IIb	+	+	+	stx1a,stx2c
151	1705722	SRR21618955	2022	Escherichia coli	O157:H7	11	CC11	IIb	+	+	+	stx1a,stx2c
152	1705755	SRR21618964	2022	Escherichia coli	O157:H7	11	CC11	IIb	+	+	+	stx1a,stx2c
153	1707787	SRR21618965	2022	Escherichia coli	O157:H7	11	CC11	IIb	+	+	+	stx1a,stx2c
154	1705709	SRR21618967	2022	Escherichia coli	O157:H7	11	CC11	IIb	+	+	+	stx1a,stx2c
155	1705694	SRR21618968	2022	Escherichia coli	O157:H7	11	CC11	IIb	+	+	+	stx1a,stx2c
156	1705724	SRR21618971	2022	Escherichia coli	O157:H7	11	CC11	IIb	+	+	+	stx1a,stx2c
157	1707835	SRR21618973	2022	Escherichia coli	O157:H7	11	CC11	IIb	+	+	+	stx1a,stx2c

This supplementary material is hosted by Eurosurveillance as supporting information alongside the article '*An outbreak of Shiga toxin-producing Escherichia coli (STEC) O157:H7 associated with contaminated lettuce and the cascading risks from climate change, the United Kingdom, August to September 2022'* on behalf of the authors, who remain responsible for the accuracy and appropriateness of the content. The same standards for ethics, copyright, attributions and permissions as for the article apply. Supplements are not edited by Eurosurveillance and the journal is not responsible for the maintenance of any links or email addresses provided therein

158	1705748	SRR21618974	2022	Escherichia coli	O157:H7	11	CC11	IIb	+	+	+	stx1a,stx2c
159	1707839	SRR21619061	2022	Escherichia coli	O157:H7	11	CC11	IIb	+	+	+	stx1a,stx2c
160	1707789	SRR21619064	2022	Escherichia coli	O157:H7	11	CC11	IIb	+	+	+	stx1a,stx2c
161	1705729	SRR21619067	2022	Escherichia coli	O157:H7	11	CC11	IIb	+	+	+	stx1a,stx2c
162	1705682	SRR21619068	2022	Escherichia coli	O157:H7	11	CC11	IIb	+	+	+	stx1a,stx2c
163	1707841	SRR21619069	2022	Escherichia coli	O157:H7	11	CC11	IIb	+	+	+	stx1a,stx2c
164	1705753	SRR21619070	2022	Escherichia coli	O157:H7	11	CC11	IIb	+	+	+	stx1a,stx2c
165	1705746	SRR21619072	2022	Escherichia coli	O157:H7	11	CC11	IIb	+	+	+	stx1a,stx2c
166	1707846	SRR21619073	2022	Escherichia coli	O157:H7	11	CC11	IIb	+	+	+	stx1a,stx2c
167	1705703	SRR21619079	2022	Escherichia coli	O157:H7	11	CC11	IIb	+	+	+	stx1a,stx2c
168	1707854	SRR21619081	2022	Escherichia coli	O157:H7	11	CC11	IIb	+	+	+	stx1a,stx2c
169	1705707	SRR21619082	2022	Escherichia coli	O157:H7	11	CC11	IIb	+	+	+	stx1a,stx2c
170	1705684	SRR21619090	2022	Escherichia coli	O157:H7	11	CC11	IIb	+	+	+	stx1a,stx2c
171	1705759	SRR21619094	2022	Escherichia coli	O157:H7	11	CC11	IIb	+	+	+	stx1a,stx2c
172	1706643	SRR21628774	2022	Escherichia coli	O157:H7	11	CC11	IIb	+	+	+	stx1a,stx2c
173	1706669	SRR21628776	2022	Escherichia coli	O157:H7	11	CC11	IIb	+	+	+	stx1a,stx2c
174	1706645	SRR21628778	2022	Escherichia coli	O157:H7	11	CC11	IIb	+	+	+	stx1a,stx2c
175	1706646	SRR21628780	2022	Escherichia coli	O157:H7	11	CC11	IIb	+	+	+	stx1a,stx2c
176	1707818	SRR21628793	2022	Escherichia coli	O157:H7	11	CC11	IIb	+	+	+	stx1a,stx2c
177	1705687	SRR21628796	2022	Escherichia coli	O157:H7	11	CC11	IIb	+	+	+	stx1a,stx2c
178	1706615	SRR21628809	2022	Escherichia coli	O157:H7	11	CC11	IIb	+	+	+	stx1a,stx2c
179	1706644	SRR21628819	2022	Escherichia coli	O157:H7	11	CC11	IIb	+	+	+	stx1a,stx2c
180	1706649	SRR21628870	2022	Escherichia coli	O157:H7	11	CC11	IIb	+	+	+	stx1a,stx2c
181	1706631	SRR21628872	2022	Escherichia coli	O157:H7	11	CC11	IIb	+	+	+	stx1a,stx2c
182	1706660	SRR21642097	2022	Escherichia coli	O157:H7	11	CC11	IIb	+	+	+	stx1a,stx2c
183	1708692	SRR21677755	2022	Escherichia coli	O157:H7	11	CC11	IIb	+	+	+	stx1a,stx2c
184	1708712	SRR21677805	2022	Escherichia coli	O157:H7	11	CC11	IIb	+	+	+	stx1a,stx2c
185	1708748	SRR21677809	2022	Escherichia coli	O157:H7	11	CC11	IIb	+	+	+	stx2c
186	1708695	SRR21677844	2022	Escherichia coli	O157:H7	11	CC11	IIb	+	+	+	stx1a,stx2c
187	1708744	SRR21677848	2022	Escherichia coli	O157:H7	11	CC11	IIb	+	+	+	stx1a,stx2c
188	1713884	SRR21910290	2022	Escherichia coli	O157:H7	11	CC11	IIb	+	+	+	stx1a,stx2c
189	1718560	SRR21910294	2022	Escherichia coli	O157:H7	11	CC11	IIb	+	+	+	stx1a,stx2c
190	1722812	SRR21910312	2022	Escherichia coli	O157:H7	11	CC11	IIb	+	+	+	stx1a,stx2c
191	1714730	SRR21910326	2022	Escherichia coli	O157:H7	11	CC11	IIb	+	+	+	stx1a,stx2c
192	1717651	SRR21910350	2022	Escherichia coli	O157:H7	11	CC11	IIb	+	+	+	stx1a,stx2c
193	1714780	SRR21910351	2022	Escherichia coli	O157:H7	11	CC11	IIb	+	+	+	stx1a,stx2c
194	1716755	SRR21910364	2022	Escherichia coli	O157:H7	11	CC11	IIb	+	+	+	stx1a,stx2c
195	1713941	SRR21910367	2022	Escherichia coli	O157:H7	11	CC11	IIb	+	+	+	stx1a,stx2c
196	1713898	SRR21910382	2022	Escherichia coli	O157:H7	11	CC11	IIb	+	+	+	stx1a,stx2c
197	1713952	SRR21910386	2022	Escherichia coli	O157:H7	11	CC11	IIb	+	+	+	stx1a,stx2c

This supplementary material is hosted by Eurosurveillance as supporting information alongside the article '*An outbreak of Shiga toxin-producing Escherichia coli (STEC) O157:H7 associated with contaminated lettuce and the cascading risks from climate change, the United Kingdom, August to September 2022'* on behalf of the authors, who remain responsible for the accuracy and appropriateness of the content. The same standards for ethics, copyright, attributions and permissions as for the article apply. Supplements are not edited by Eurosurveillance and the journal is not responsible for the maintenance of any links or email addresses provided therein

198	1713907	SRR21910387	2022	Escherichia coli	O157:H7	11	CC11	IIb	+	+	+	stx1a,stx2c
199	1721273	SRR21910417	2022	Escherichia coli	O157:H7	11	CC11	IIb	+	+	+	stx1a,stx2c
200	1726583	SRR21910585	2022	Escherichia coli	O157:H7	11	CC11	IIb	+	+	+	stx1a,stx2c
201	1721237	SRR21910590	2022	Escherichia coli	O157:H7	11	CC11	IIb	+	+	+	stx1a,stx2c
202	1725324	SRR21910671	2022	Escherichia coli	O157:H7	11	CC11	IIb	+	+	+	stx1a,stx2c
203	1714770	SRR21910686	2022	Escherichia coli	O157:H7	11	CC11	IIb	+	+	+	stx1a,stx2c
204	1717657	SRR21910688	2022	Escherichia coli	O157:H7	11	CC11	IIb	+	+	+	stx1a,stx2c
205	1717588	SRR21910690	2022	Escherichia coli	O157:H7	11	CC11	IIb	+	+	+	stx1a,stx2c
206	1713946	SRR21910747	2022	Escherichia coli	O157:H7	11	CC11	IIb	+	+	+	stx1a,stx2c
207	1714761	SRR21910781	2022	Escherichia coli	O157:H7	11	CC11	IIb	+	+	+	stx1a,stx2c
208	1710439	SRR21918425	2022	Escherichia coli	O157:H7	11	CC11	IIb	+	+	+	stx1a,stx2c
209	1711721	SRR21918429	2022	Escherichia coli	O157:H7	11	CC11	IIb	+	+	+	stx1a,stx2c
210	1713959	SRR21918445	2022	Escherichia coli	O157:H7	11	CC11	IIb	+	+	+	stx1a,stx2c
211	1710426	SRR21918456	2022	Escherichia coli	O157:H7	11	CC11	IIb	+	+	+	stx1a,stx2c
212	1711724	SRR21918457	2022	Escherichia coli	O157:H7	11	CC11	IIb	+	+	+	stx1a,stx2c
213	1711738	SRR21918462	2022	Escherichia coli	O157:H7	11	CC11	IIb	+	+	+	stx1a,stx2c
214	1711766	SRR21918478	2022	Escherichia coli	O157:H7	11	CC11	IIb	+	+	+	stx1a,stx2c
215	1711762	SRR21918490	2022	Escherichia coli	O157:H7	11	CC11	IIb	+	+	+	stx1a,stx2c
216	1711741	SRR21918520	2022	Escherichia coli	O157:H7	11	CC11	IIb	+	+	+	stx1a,stx2c
217	1710388	SRR21918521	2022	Escherichia coli	O157:H7	11	CC11	IIb	+	+	+	stx1a,stx2c
218	1711737	SRR21918546	2022	Escherichia coli	O157:H7	11	CC11	IIb	+	+	+	stx1a,stx2c
219	1710394	SRR21918585	2022	Escherichia coli	O157:H7	11	CC11	IIb	+	+	+	stx1a,stx2c
220	1710384	SRR21918587	2022	Escherichia coli	O157:H7	11	CC11	IIb	+	+	+	stx1a,stx2c
221	1710437	SRR21934061	2022	Escherichia coli	O157:H7	11	CC11	IIb	+	+	+	stx1a,stx2c
222	1733208	SRR22016569	2022	Escherichia coli	O157:H7	11	CC11	IIb	+	+	+	stx1a,stx2c
223	1736120	SRR22069848	2022	Escherichia coli	O157:H7	11	CC11	IIb	+	+	+	stx1a,stx2c
224	1735021	SRR22085145	2022	Escherichia coli	O157:H7	11	CC11	IIb	+	+	+	stx1a,stx2c
225	1738048	SRR22096621	2022	Escherichia coli	O157:H7	11	CC11	IIb	+	+	+	stx1a,stx2c
226	1740471	SRR22148707	2022	Escherichia coli	O157:H7	11	CC11	IIb	+	+	+	stx1a,stx2c
227	1746694	SRR22247558	2022	Escherichia coli	O157:H7	11	CC11	IIb	+	+	+	stx1a,stx2c
228	1746695	SRR22247600	2022	Escherichia coli	O157:H7	11	CC11	IIb	+	+	+	stx1a,stx2c
229	1752770	SRR22314601	2022	Escherichia coli	O157:H7	11	CC11	IIb	+	+	+	stx1a,stx2c

This supplementary material is hosted by Eurosurveillance as supporting information alongside the article '*An outbreak of Shiga toxin-producing Escherichia coli (STEC) O157:H7 associated with contaminated lettuce and the cascading risks from climate change, the United Kingdom, August to September 2022'* on behalf of the authors, who remain responsible for the accuracy and appropriateness of the content. The same standards for ethics, copyright, attributions and permissions as for the article apply. Supplements are not edited by Eurosurveillance and the journal is not responsible for the maintenance of any links or email addresses provided therein

Supplementary Table S2. Univariable associations between exposure and case status for any variable forming part of the null hypotheses or with any evidence of a positive association with the outcome (odds ratio greater than 1, p-value <0.20)

Exposure	Cases			Controls			Odds ratio	95% confidence interval		LRT p-value
	Total	Exposed	%	Total	Exposed	%		Lower	Upper	
**Any chicken**	41	40	98	206	152	74	9.65	2.48	87.0	<0.001
Chicken pieces	39	13	33	205	38	19	2.22	1.03	4.61	0.04
Chicken nuggets/ popcorn chicken	40	12	30	204	38	19	1.90	0.87	3.96	0.10
Chicken wrap	40	7	18	204	21	10	1.91	0.73	4.59	0.18
Chicken goujons	39	6	15	203	17	8	2.07	0.73	5.27	0.16
**Any salad leaves**	41	34	83	206	133	65	2.53	1.15	6.29	0.02
Lettuce	41	32	78	206	127	62	2.13	1.02	4.87	0.05
Iceberg lettuce	33	22	67	185	72	39	3.06	1.44	6.81	0.003
**Any beef**	41	29	71	206	104	50	2.31	1.16	4.88	0.02
Other beef item	41	2	5	205	1	0	8.67	1.12	96.63	0.04

This supplementary material is hosted by Eurosurveillance as supporting information alongside the article '*An outbreak of Shiga toxin-producing Escherichia coli (STEC) O157:H7 associated with contaminated lettuce and the cascading risks from climate change, the United Kingdom, August to September 2022'* on behalf of the authors, who remain responsible for the accuracy and appropriateness of the content. The same standards for ethics, copyright, attributions and permissions as for the article apply. Supplements are not edited by Eurosurveillance and the journal is not responsible for the maintenance of any links or email addresses provided therein

Supplementary Table S3. Multivariable analysis (model 1) containing composite variables (n=247; 41 cases and 206 controls).

Variable	Category	Adjusted OR	95% confidence interval		p-value	LRT p-value
			Lower	Upper		
Any chicken	No chicken eaten	Reference				<0.001
	Eaten only prepared at home	2.33	0.32	16.86	0.39	
	Eaten prepared away from home*	12.73	2.24	72.16	<0.001	
Any salad leaves	No salad leaves eaten	Reference				0.20
	Eaten prepared at home or away	1.76	0.72	4.31	0.20	
Any beef	No beef eaten	Reference				0.29
	Eaten prepared at home or away	1.53	0.69	3.38	0.29	
Age group	0-19 years	Reference				0.28
	20-39 years	0.82	0.33	2.04	0.67	
	>40 years	1.70	0.59	4.96	0.33	
Gender	Female	Reference				0.28
	Male	0.99	0.48	2.03	0.98	
	Other	13.3	0.41	434.23	0.11	
Country	England	Reference				0.99
	Northern Ireland	0.90	0.23	3.51	0.88	
	Scotland	0.85	0.27	2.73	0.79	
	Wales	0.87	0.27	2.82	0.81	
Proton pump inhibitor	Not known to have taken medication	Reference				0.43
	Took medication	1.55	0.53	4.50	0.43	

This supplementary material is hosted by Eurosurveillance as supporting information alongside the article '*An outbreak of Shiga toxin-producing Escherichia coli (STEC) O157:H7 associated with contaminated lettuce and the cascading risks from climate change, the United Kingdom, August to September 2022'* on behalf of the authors, who remain responsible for the accuracy and appropriateness of the content. The same standards for ethics, copyright, attributions and permissions as for the article apply. Supplements are not edited by Eurosurveillance and the journal is not responsible for the maintenance of any links or email addresses provided therein

Supplementary Table S4. Multivariable analysis (model 2) containing product and ingredient level variables, constructed following investigation of individual salad and chicken items (n=214; 30 cases and 184 controls).

Variable	Category	Adjusted OR	95% confidence interval		p-value*	LRT p-value
			Lower	Upper		
Chicken pieces	Not eaten chicken or eaten only at home	Reference				0.005
	Eaten prepared away from home**	5.1	1.65	16.14	0.005	
Chicken goujons	Not eaten chicken or eaten away from home	Reference				0.064
	Eaten only prepared at home	5.34	0.91	30.75	0.06	
Chicken nuggets/ popcorn chicken	Not eaten chicken nuggets/popcorn chicken	Reference				0.41
	Eaten prepared at home or away	1.57	0.53	4.48	0.41	
Iceberg lettuce	Not eaten iceberg lettuce	Reference				0.009
	Eaten prepared at home or away	3.38	1.35	9.23	0.009	
Any beef	Not eaten any beef	Reference				0.18
	Eaten prepared at home or away	1.94	0.73	5.57	0.18	
Age group	<20 years	Reference				0.41
	20-39 years	1.11	0.36	3.73	0.86	
	40+ years	2.31	0.56	11.03	0.25	
Gender	Female	Reference				0.05
	Male	2.00	0.82	5.1	0.13	
	Other	32.07	1.15	5614.79	0.04	
Country	England	Reference				0.97
	Northern Ireland	0.73	0.08	4.2	0.75	
	Scotland	0.76	0.16	2.77	0.69	
	Wales	0.82	0.19	2.88	0.77	
Proton pump inhibitor	Not known to have taken medication	Reference				0.43
	Took medication	1.73	0.41	6.15	0.43	

This supplementary material is hosted by Eurosurveillance as supporting information alongside the article '*An outbreak of Shiga toxin-producing Escherichia coli (STEC) O157:H7 associated with contaminated lettuce and the cascading risks from climate change, the United Kingdom, August to September 2022*' on behalf of the authors, who remain responsible for the accuracy and appropriateness of the content. The same standards for ethics, copyright, attributions and permissions as for the article apply. Supplements are not edited by Eurosurveillance and the journal is not responsible for the maintenance of any links or email addresses provided therein
